# A cost-utility analysis of a rehabilitation service for people living with and beyond cancer

**DOI:** 10.1186/s12913-014-0558-5

**Published:** 2014-11-19

**Authors:** Jeff Round, Baptiste Leurent, Louise Jones

**Affiliations:** Marie Curie Palliative Care Research Unit, University College London, London, UK; University College London Comprehensive Clinical Trials Unit, University College London, Gower Street, London, WC1E 6BT UK

**Keywords:** Cost-utility, Economics, Health economics, Survivorship, Living beyond cancer

## Abstract

**Background:**

We conducted a wait-list control randomised trial of an outpatient rehabilitation service for people living with and beyond cancer, delivered in a hospice day care unit. We report the results of an economic evaluation undertaken using the trial data.

**Methods:**

Forty-one participants were recruited into the study. A within-trial stochastic cost-utility analysis was undertaken using Monte-Carlo simulation. The outcome measure for the economic evaluation was quality adjusted life years (QALYs). Costs were measured from the perspective of the NHS and personal social services. Uncertainty in the observed data was captured through probabilistic sensitivity analysis. Scenario analysis was conducted to explore the effects of changing the way QALYs were estimated and adjusting for baseline difference in the population. We also explore assumptions about the length of treatment benefit being maintained.

**Results:**

The incremental cost-effectiveness ratio (ICER) for the base-case analysis was £14,231 per QALY. When QALYs were assumed to change linearly over time, this increased to £20,514 per QALY at three months. Adjusting the estimate of QALYs to account for differences in the population at baseline increased the ICER to £94,748 per QALY at three months. Increasing the assumed length of treatment benefit led to reduced ICERs in all scenarios.

**Conclusions:**

Although the intervention is likely to be cost-effective in some circumstances, there is considerable uncertainty surrounding the decision to implement the service. Further research, informed by a formal value of information analysis, would reduce this uncertainty.

## Background

More than 2 million people across the UK are currently living with and beyond cancer. For those who have recently completed active treatments, but for whom cure remains unlikely, there may be considerable physical and psychological morbidity [1,2]. Increasingly there is a trend towards providing palliative care to those who might still be considering active treatments from oncology services to improve symptom control [3,4]. It remains common practice to refer people with cancer to palliative care specialists when standard therapy has failed and cure is no longer likely. At the point of referral estimating prognosis may be difficult [5]. Patients may typically access the service for more than three months and the care that is offered is adjusted according to deteriorating health status. Attendance at day care services may enable a smooth transition to terminal care either as a hospice in patient or at home. A rehabilitation-focussed approach to care aims to improve quality of survival, helping people adapt to current circumstances, lead fulfilling lives and function at a minimum level of dependency regardless of life expectancy [6]. For those with active recurrent progressive disease, many UK hospices provide out-patient specialist palliative day therapy. Such care is increasingly offered to not only to people with cancer but to those with a range of other advanced, progressive diseases who are approaching the end of their lives.

Despite a common focus on provision of individually tailored holistic care, there is variability in the interventions available, ranging from social support to a clinically oriented service [7]. Specialist palliative care provision in hospices within the UK is largely dependent on funding spearheaded by the voluntary sector and what is available in any locality will depend on local resources and availability of staff, premises and equipment. More specialised services rely on the availability of a multi-disciplinary team with the necessary skills and training. Outpatient clinical assessment may be offered by doctors or clinical nurse specialists. Such consultations may be used to identify particular supportive care needs and to agree a care plan that might include sessions delivered by physiotherapists, counsellors or complementary therapists. In addition, some attendees may benefit from the support of interaction with others who are faced with similar health challenges or from group activities such as relaxation or guidance for the management of symptoms such as shortness of breath [8].

There is little current evidence on the relative effectiveness of different models of day therapy, and little guidance to inform those setting up new services or considering how best to use limited available resource for the maximum benefit of patients [9,10]. Recent reports that review available qualitative data suggest a positive impact on quality of life for people receiving hospice day care [11]. However data reporting the effects on quantitative outcomes are scarce, in part due to the complexity of services offered and uncertainty over the choice of appropriate measures [12,13]. Whilst there is some evidence from a study conducted in 5 centres in SE England [14] that those receiving hospice day care used fewer other health care services, there is a lack of research to assess the costs and cost-effectiveness of hospice day care services [15].

Outpatient services provided by hospices as described above are considered by some to be an effective way of providing care to patients that addresses both the physical and psychological needs of patients. We conducted an RCT that showed an outpatient service was capable of addressing these needs. We also investigated whether such a service is cost-effective and this is the focus of the current report. In reporting the clinical results of the trial [6] we were restricted by space to describing a limited economic analysis of the data available at the primary outcome point of three months from randomisation (these results are reproduced here in Table 1). In this study we report in full the methodology and results of the economic evaluation, in the form of a cost utility analysis with full probabilistic sensitivity analysis and scenario analyses.

**Table 1:** **Incremental cost-effectiveness ratio (reproduced from Jones et al., 2012** [16])

**Control**		**Intervention**		**Incremental**		
**Cost (95% CI)**	**QALYs (95% CI)**	**Cost (95% CI)**	**QALYs (95% CI)**	**Cost (95%CI)**	**QALYs (95%CI)**	**ICER**
£1,590	0.11	£2,544	0.16	£955	0.05	£19,391
(£1,129 - £2,193)	(0.058 - 0.157)	(£1,890 - £3,482)	(0.137 - 0.187)	(£82 - £1,975)	(0.000 – 0.112)	

## Methods

This analysis is based on data collected as part of a completed randomised controlled trial of the effectiveness of a complex rehabilitation intervention in a DTU for people with advanced, progressive, recurrent cancer [16]. Ethical approval for the trial was received from the joint University College London/University College London Hospital (UCLH) Research Ethics Committee on October 5, 2009, ref. 09/H0714/46. The trial was registered with ISRCTN number 22485853. The primary outcome of the trial was psychological subscale of the Supportive Care Needs Survey (SCNS) [17].

Data were collected as part of a two-arm, wait-list, randomised controlled trial of the rehabilitation service in addition to usual care compared with usual care alone [16]. Forty-one patients with active, progressive, recurrent malignancies were recruited from breast and haematological oncology out-patient clinics at the Royal Free Hospital (RFH) and University College London Hospital (UCLH) joint cancer centre London UK, between August 2010 and July 2011. Ninety-three per cent were female and the median age was 62 years. Thirty-six participants (20 in the intervention arm, and 16 in the control group) completed the 3-month follow-up for whom both effectiveness and economic outcomes were available. Reasons for drop out were independent of the intervention and analysis was performed on complete data.

### The rehabilitation intervention

The rehabilitation intervention was developed and defined using an iterative “plan-do-study-act” approach consistent with the guidance on developing complex healthcare interventions outlined by the UK Medical Research Council [18]. It offers individual clinical assessment, agreed goal setting and a complex package of specialist multi-disciplinary services including physical and psychological therapies. The aim is to achieve an agreed date for discharge from the service, usually within 3–6 months. Those whose health continues to deteriorate may not achieve discharge. Details of the intervention are described in the form of a written manual, available from the authors.

### Effectiveness of the intervention

The effectiveness of this intervention was evaluated in a wait-list randomised controlled trial conducted in a large inner city cancer centre and the results are published elsewhere [16]. For the trial we chose participants with active recurrent progressive breast and haematological cancers because post treatment needs are well documented for these disease sites. We found that the outpatient day-therapy rehabilitation approach was significantly more effective than usual care alone for the primary outcome of psychological needs for care as measured by the Supportive Care Needs Survey [17]. Health-related quality of life measured using the EQ-5D was also found to be higher in the treatment group, although this difference was not significant and the trial was not powered to detect a difference on the quality of life outcome measure.

### Cost-effectiveness of the intervention

The trial was limited to a three month data collection phase due to the wait list design, ethical constraints and restrictions on research funding. In this paper we develop more fully the economic evaluation to a) include a full probabilistic sensitivity analysis and b) test assumptions made about treatment benefit in the deterministic analysis. We test these assumptions through scenario analyses focusing on i) the methods used to calculate quality adjusted life years and ii) the extrapolation of treatment costs and benefits beyond the initial three month follow-up period. Our updated results provide additional information on the likely longer term impact of the intervention. This is of importance as people with advanced disease are likely to have on-going supportive care needs and many will remain within the service for an extended period. For those that achieve discharge from the service, it is hoped that any benefits accrued would be maintained.

A cost-utility analysis of the intervention compared with usual care was conducted. The main outcome of interest for the cost-utility analysis was quality of life, expressed as quality adjusted life years (QALYs) and measured using the EQ-5D. The EQ-5D is a commonly used instrument for the measurement of health related quality of life [19] and is recommended for use in technology appraisal by the National Institute for Health and Care Excellence [20]. Although prognosis of participants was limited, minimum dependency and maximum function were key components of the primary outcome and we therefore considered the EQ-5D to be the most appropriate preference based measure of HRQoL [21]. The analysis was undertaken from both an NHS and a personal social services perspective. Results are reported as incremental cost-effectiveness ratios (ICERs) and cost-effectiveness acceptability curves (CEACs). A CEAC shows the percentage of simulations in which an intervention is cost-effective (based on the results of the Monte Carlo simulations) across a range of willingness to pay per QALY. Costs and benefits were estimated for the three month trial period in the analysis, thus no discount rate was applied. Prices are given in UK pounds at 2010–2011 values.

The base-case analysis presented is a stochastic cost-effectiveness analysis [22]. A Monte Carlo process modelling 10,000 simulations was performed following the approach established by O’Hagan and colleagues [23-25]. Parameter uncertainty was reflected in the analysis through the use of probabilistic sensitivity analysis (PSA) in a Bayesian framework. Due to the limited data available, an uninformative prior was assumed [26]. PSA is used to reflect the uncertainty inherent in input parameters estimated from samples of populations. Model parameters are characterised not as point estimates based on measures of central tendency but as probability distributions. For each simulation in a PSA, an estimate for each parameter is drawn from the assigned distribution. The individual values sampled from the distributions are then used to estimate the results of the model for that simulation. The results of all simulations are then combined to give an overall result. Details of the mean values and probability distributions estimated for each parameter are presented in Table 2.

**Table 2:** **Resource use and utility estimates used in economic evaluation (estimated from trial data)**

	**Control**			**Intervention**		
	**Mean (s** ^**2**^ **)**	**Distribution**	**(alpha, beta)**	**Mean (s** ^**2**^ **)**	**Distribution**	**(alpha, beta)**
Utility values	0.447 (0.01)	Gamma	(29.45, 0.02)	0.654 (0.002)	Gamma	(48.01, 0.01)
Outpatient appointment	5.75 (1.00)	Gamma	(32.95, 0.17)	5.16 (0.44)	Gamma	(61.08, 0.08)
GP appointment	1.56 (0.25)	Gamma	(9.65, 0.16)	1.00 (0.10)	Gamma	(10.50, 0.10)
Other NHS services	1.75 (0.39)	Gamma	(7.85, 0.22)	0.32 (0.03)	Gamma	(3.90, 0.08)
Counsellor	0.94 (0.34)	Gamma	(2.56, 0.37)	Not applicable	Not applicable	Not applicable
Physiotherapist	1.81 (1.24)	Gamma	(2.65, 0.68)	Not applicable	Not applicable	Not applicable
Outpatient Hospice	Not applicable	Not applicable	Not applicable	2.05 (0.07)	Gamma	(59.26, 0.03)
DTU Attendance	Not applicable	Not applicable	Not applicable	5.20 (1.05)	Gamma	(25.74, 0.20)
Hospice Physiotherapy	Not applicable	Not applicable	Not applicable	3.55 (0.97)	Gamma	(12.98, 0.27)
Other hospice services	Not applicable	Not applicable	Not applicable	1.5 (0.21)	Gamma	(10.82, 0.14)

At three months, participants reported their use of medical services since baseline using a modified version of the Client Service Receipt Inventory CSRI [27]. We collected data primarily on their use of NHS and hospice services. Commonly accessed services included general practitioners, physiotherapists, psychologists or other counsellors, outpatient hospital appointments. Other services were less frequently accessed and so have been aggregated in the analysis, weighted according to frequency of access and cost (see Table 2).

Data on costs were derived from the Unit Costs of Health and Social Care [28]; where publicly available data were not available, unit costs were obtained from the hospice where the trial was conducted. The primary source for unit cost data [28] does not provide estimates of variability in unit costs. To reflect variation in unit costs, an adjustment is made to the total cost estimated for each simulation using the market forces factor used to calculate local adjustments to NHS tariff prices. An upper and lower bound for a uniform distribution was defined to reflect the market forces factor [29]. For each simulation, a sample from this distribution was used to weight the expected total cost per patient. Sources for all unit costs are presented in Table 3. The way in which variability in costs was accounted for in the model is the underlying reason that the results presented in Jones et al. [16] differ from the base-case results presented here. In our previous estimate, costs were not included in the probabilistic sensitivity analysis. We consider the current analysis reported here to be more robust than the previous analysis.

**Table 3:** **Cost estimates used in economic evaluation**

**Resource**	**Mean cost/unit**	**Unit**	**Resource**	**Mean cost/unit**	**Unit**
Outpatient appointment	£152.00 [28]	Outpatient attendance	Counsellor	£44.00 [28]	Per hour client contact
GP appointment	£32.00 [28]	per 12 minute consultation	Inpatient admission	£225.00 [28]	NHS cost per bed day
GP home visit	£115.00 [28]	25 minute visit (£4.60 per minute)	Outpatient Hospice	£104.40*	Per hospice outpatient appt
Practice Nurse	£10.00 [28]	Per consultation	DTU Attendance	£139.80*	Cost per DTU attendance
District nurse (home)	£68.00 [28]	Per hour home visit	Hospice Physiotherapist	£18.50 [28]	30 minute consultation
Palliative nurse (home)	£68.00 [28]	Per hour home visit	Hospice Other	£37.27 [28]*	Weighted average of hospice services used by the intervention group
Occupational therapist	£51.33 [28]	40 minute consultation	Control group other services	£66.09 [28]*	Weighted average of other service use costs
Physiotherapist	£18.50 [28]	30 minute consultation	Intervention group other services	£70.42 [28]*	Weighted average of other service use costs
Psychologist	£81.00 [28]	Per hour client contact			

*Costs provided by the hospice participating in the study.

Utility values were estimated using the EQ-5D social tariff for the UK [19] with scores estimated at baseline and at three month follow-up. In the base-case analysis as reported elsewhere [16] QALYs were initially calculated using the a crude mean difference between the intervention and control group at three months. We test this assumption in scenario analyses as described below.

### Scenario analyses

We conducted a series of scenario-based sensitivity analyses to explore variation in our results that arise owing to changes in assumptions made for the base-case analysis. We explored variations around two key assumptions. The first is the manner in which we calculated QALYs from the trial data. The second assumption we test is the length of time over which benefit is maintained following treatment.

In the analysis reported alongside the clinical trial [16] we used a crude estimate of benefit, where we simply calculated the treatment effect in QALYs based on the difference in utility scores between treatment groups at three months follow-up. This has the effect of allocating all benefit arising from treatment immediately at the start of treatment. While this may be appropriate in some scenarios, for this rehabilitation intervention it is likely that this overstates any differences in QALYs between groups at three months. We therefore explore two alternative approaches to calculating QALYs.

In the first sensitivity scenario QALYs were calculated by integrating utility values over time (area under the curve), based on EQ-5D values at baseline and three months, and assuming a linear change between measurements [30]. This approach assumes that changes in outcome accrue gradually over time. We thereafter refer to this scenario as “area under the curve approach”.

We then adjusted the estimate of QALY difference between groups to account for differences at baseline. This second approach takes into account the imbalance between groups in EQ5D at baseline and how this could affect the observed difference in QALYs [30]. It can also increase precision by reducing standard errors of the difference in QALY estimates. However the adjustment is based on a linear regression and assumes a normal distribution of the difference in QALYs between groups, which may not always hold in a small sample. We thereafter refer to this scenario as “baseline adjusted approach”.

The final scenario we tested was the length of time over which patients are assumed to derive benefit from the intervention. The base-case analysis considered only the period during which patients were active trial participants. However, an important component of the intervention is that it aims to enable patients to be discharged from the service, or to reduce their frequency of attendance through agreed goal-setting. It is expected that benefit would be maintained for some period of time following discharge. However, the limited time frame of the trial follow-up period prevented the collection of data on patient outcomes following discharge. To test what might happen, we considered the benefit of treatment being maintained over three, six and nine months beyond completion of the follow-up.

Further assumptions were needed about how to estimate benefits at future time points in the absence of follow-up data beyond three months. We have assumed that the mean difference in outcomes at the three month follow-up point was maintained at future time points. Although it is unlikely that in practice benefit would be maintained at a constant level between three month follow-up and one year, there is no evidence on which to base any additional assumption. Our current assumption makes no prediction about changes in quality of life following treatment; rather we assume only that the difference between the treatment and control groups is maintained. We consider the implications of this assumption in more detail in the discussion. We also assume that no additional differences in cost arise during the extended period of analysis. While it is possible that the intervention could influence resource use beyond the three month trial period we expect most of the difference to happened in the first three months when patients were attending the day therapy unit (DTU). It is unknown in which direction any longer term difference would be and we therefore made the assumption of no difference in resource use between arms after the initial three month period.

**Figure 1:**
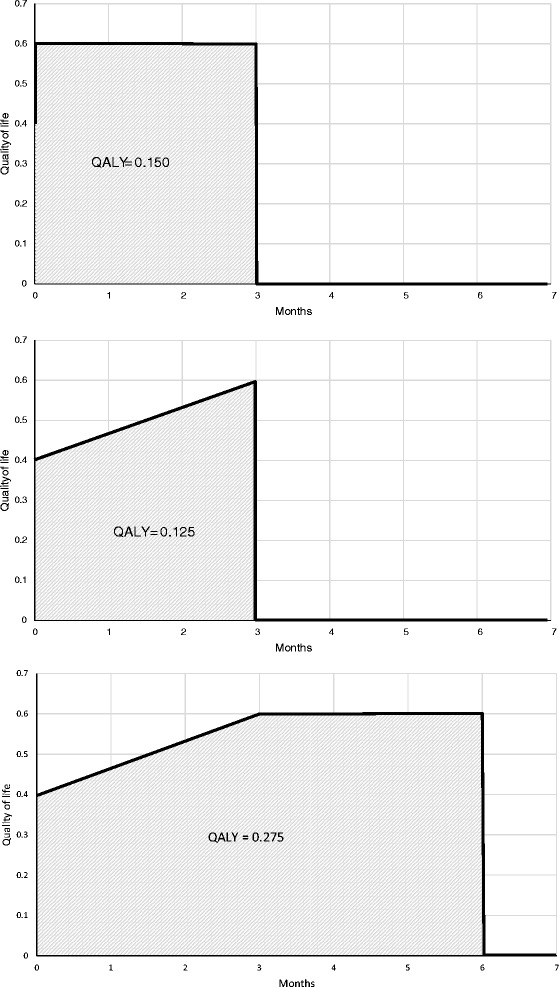
**Example of different approaches for calculating QALYs.**

Figure 1 illustrates how different approaches to estimating patient benefit can lead widely differing results in QALYs used in an analysis.

## Results

### Resource use

In Table 4 we show a summary of the mean cost for the key resource use categories for the control and intervention arms. While the study was not powered to detect differences in resource use, the descriptive results clearly show that the primary difference in expected cost arises from attendance at hospice outpatient and day therapy units. These cost components form part of the intervention and were not available to the control group.

**Table 4:** **Deterministic mean cost of individual resources**

	**Expected cost**	
**Resource**	**Control**	**Intervention**
Outpatient appointment	£874	£784
GP appointment	£50	£32
Combined other	£101	£58
Physiotherapist	£34	Not applicable
Counsellor	£41	Not applicable
Outpatient hospice	Not applicable	£214
DTU Attendance	Not applicable	£727
Total expected cost	£1,100	£1,775

### Base-case analysis

Over the trial period of 3 months, the expected mean difference in cost from the Monte Carlo simulation in the base-case analysis was £735 (95% Bayesian credible intervals (CI) £221 to £1,271) and the mean difference in QALYs was 0.052 (95% CI 0.040 to 0.063). The ICER of the mean incremental values is £14,231 per QALY (Table 5).

**Table 5:** **Base-case results (with Bayesian credible intervals)**

	**Control**		**Intervention**		**Incremental**		
**Base-case**	**Cost**	**QALYs**	**Cost**	**QALYs**	**Cost**	**QALYs**	**ICER**
	**(95% CI)**	**(95% CI)**	**(95% CI)**	**(95% CI)**	**(95% CI)**	**(95% CI)**	**£s/QALY**
Benefit as measured at three months	£1,193	0.112	£1,928	0.164	£735	0.052	£14,231
	(£840 to £1,638)	(0.102 to 0.123)	(£1,481 to £2,455)	(0.159 to 0.168)	(£221 to £1,271)	(0.040 to 0.063)	

### Scenario analyses

**Figure 2:**
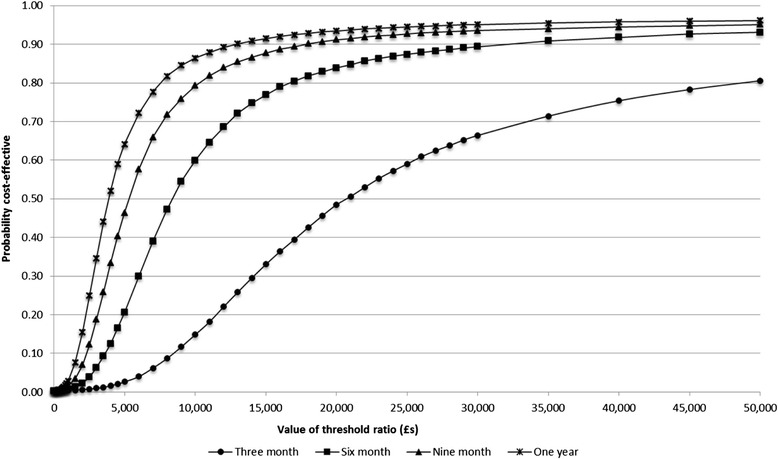
**CEACs of rehabilitation intervention compared to usual care for unadjusted QALY estimates.**

The results of the scenario analyses are presented in Tables 5 and 6 as well as Figures 1 and 2. Using the area under the curve approach to estimating QALYs then the intervention is expected to be cost-effective in 42.9% and 64.1% of simulations at threshold values of £20,000 and £30,000 respectively at three months, with an ICER of £20,514 (Table 6). The likelihood of the intervention being cost-effective increases with the length of time benefit is expected to be maintained. It rises to 77.4% at a threshold value of £20,000 when benefit is measured at six months. If benefit is maintained over 12 months then at a threshold value of £20,000 the intervention is expected to be cost-effective in 89.6% of simulations (Figure 2).

**Table 6:** **Area under the curve scenario analysis (with Bayesian credible intervals)**

	**Control**		**Intervention**	**ICER**	
**Area under the curve analysis**	**Cost**	**QALYs**	**Cost**	**QALYs**	**£s/QALY**
	**(95% CI)**	**(95% CI)**	**(95% CI)**	**(95% CI)**	
Benefit as measured at three months	£1,202	0.120	£1,936	0.156	£20,514
	(£842 to £1,631)	(0.077 to 0.160)	(£1,483 to £2,459)	(0.136 to 0.176)	
Benefit maintained to six months	£1,192	0.231	£1,931	0.312	£8,371
	(£837 to £1,630)	(0.138 to 0.318)	(£1,471 to £2,477)	(0.267 to 0.355)	
Benefit maintained to nine months	£1,196	0.344	£1,931	0.470	£5,224
	(£832 to £1,626)	(0.199 to 0.475)	(£1,481 to £2,460)	(0.398 to 0.537)	
Benefit maintained to one year	£1,198	0.454	£1,931	0.629	£3,815
	(£843 to £1,623)	(0.247 to 0.623)	(£1,476 to £2,469)	(0.526 to 0.718)

**Figure 3:**
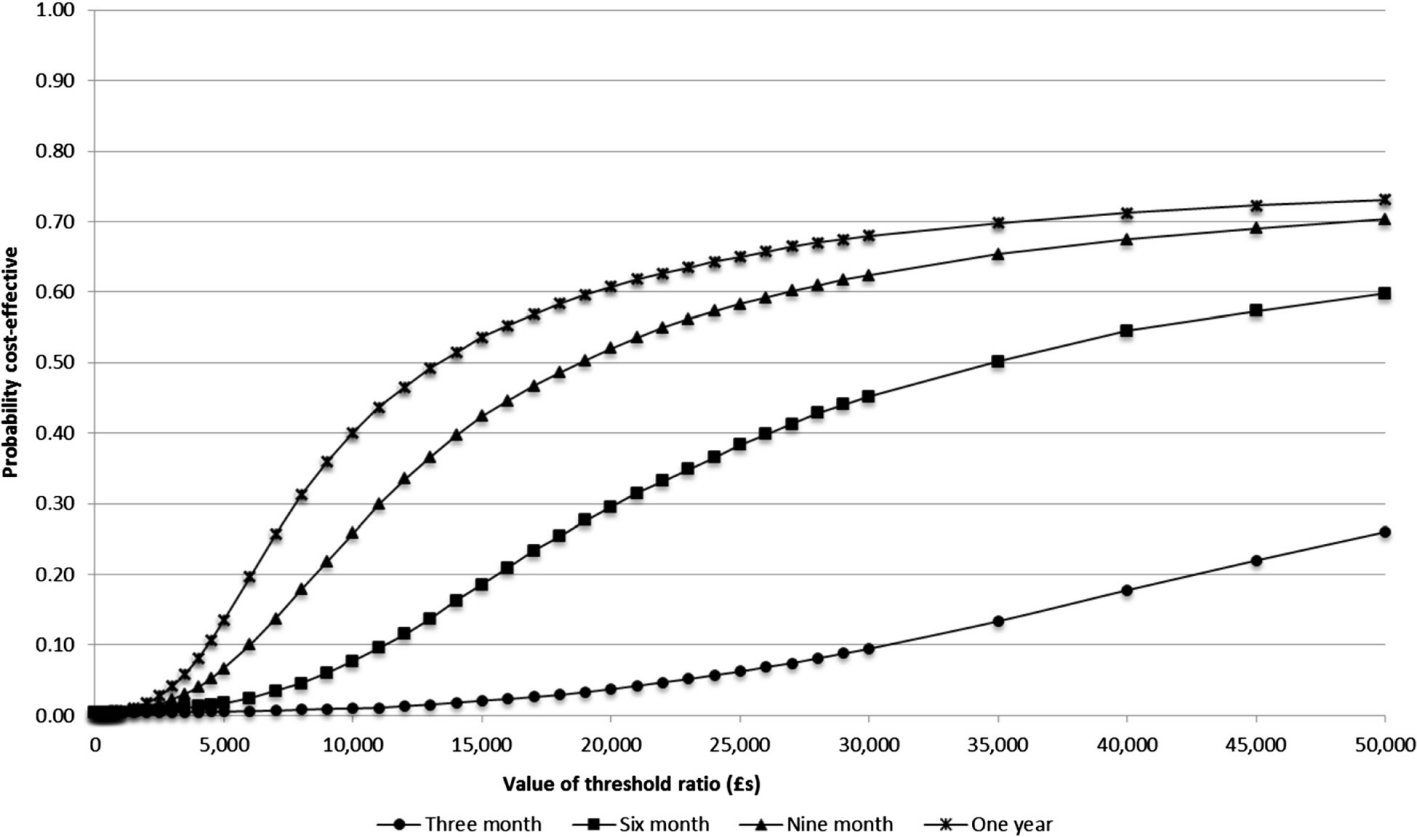
**CEACs of rehabilitation intervention compared to usual care for adjusted QALY estimates.**

When we calculate QALYs adjusted for baseline differences in the control and intervention population, the intervention becomes less likely to be cost-effective across all time-horizons and threshold values. The estimated ICER at three months is £94,748 (Table 7) and consequently the intervention is highly unlikely to be cost-effective when benefit is only gained for three months, with a probability of being cost-effective of just 3.7% at the £20,000 threshold and 9.5% at the higher £30,000 threshold (Figure 3). If benefit is maintained for a total of six months then the intervention reaches a probability of being cost effective of 29.5% and 45.2% as threshold values of £20,000 and £30,000. Only where benefit is maintained for nine months or longer is the intervention more likely than not to be cost-effective at a threshold value of £20,000 per QALY. At nine months the intervention is cost-effective in 50.2% of simulations and at 12 months 60.8% of simulations at a threshold value of £20,000.

**Table 7:** **Baseline adjusted scenario analysis (with Bayesian credible intervals)**

	**Costs**		**Estimated QALY difference**		**ICER**
	**(95%CI)**				
**Baseline adjusted analysis**	**Control**	**Intervention**	**Mean**	**SE**	**£s/QALY**
Benefit as measured at three months	£1,203	£1,938	0.008	0.009	£94,748
	(£865 to £1,615)	(£1,540 to £2,394)	---	---	
Benefit maintained to six months	£1,202	£1,931	0.023	0.027	£29,835
	(£837 to £1,658)	(£1,471 to £2,437)	---	---	
Benefit maintained to nine months	£1,207	£1,932	0.039	0.046	£18,771
	(£868 to £1,626)	(£1,534 to £2,389)	---	---	
Benefit maintained to one year	£1,205	£1,933	0.054	0.064	£13,400
	(£864 to £1,611)	(£1,540 to £2,389)	---	---

## Discussion

We report details of a full cost-utility analysis and associated sensitivity analyses of a rehabilitation intervention that has been shown to be clinically effective in a single-centre randomised controlled trial. Our results demonstrate two things. First, that the results of the analysis are sensitive to the method used to estimate QALYs. This is well supported in the literature (for a discussion of this, see Manca and colleagues [30]). Second, it is also clear that the longer treatment benefit is maintained, the more likely it becomes that the intervention (which is provided over a short period of time) represents a cost-effective use of resources. The CEACs in Figures 1 and 2 are used to show in what proportion of the Monte-Carlo simulations the intervention is expected to have a greater net-benefit compared with control across a range of threshold values of the decision maker’s willingness to pay for a QALY [31]. In England, for the NHS NICE typically use a threshold range of £20,000 to £30,000 per QALY when determining whether a treatment is cost-effective use of resources.

Our results suggest that a standardised hospice based day therapy intervention has the potential to be cost-effective, though in the base-case analysis this effect was marginal with respect to the NICE threshold value of £20,000 per QALY in England [32]. We have shown that when QALYs are estimated using the more robust area-under-the-curve or baseline-adjusted approaches then the intervention is less likely to be cost-effective at three months than in our initial crude analysis. However, if the benefit of the intervention were maintained as intended following discharge from the service, then the likelihood of the intervention being cost-effective clearly increases; as expected, the longer any observed benefit is maintained post-treatment, the more likely the intervention is a cost-effective use of resources. This finding is independent of the method used to estimate QALYs.

Although an RCT provides a robust causal estimate of the intervention effect, a single trial may not provide sufficient evidence on which to base a decision on cost effectiveness [33]. For example, trials such as this which are limited to single centres, small sample sizes or restrictions on clinical diagnoses such as cancer types may not show to what extent the effectiveness of an intervention is transferable to other settings or patient populations. In addition trial designs, such as wait-list control, may not include an appropriate time horizon for the measurement of treatment costs and outcomes, and may not be designed to consider all relevant treatment alternatives [33]. Such limitations are often pragmatic and reflect funding restrictions or clinical circumstances. In this case, caution was exercised due to the ethics of withholding a potentially beneficial supportive intervention for those with a limited prognosis.

### Transferability of results

In addition to the consideration of local factors and context that influence treatment outcomes in a single centre study, interpreting economic results also requires consideration of patterns of resource use and costs associated with a single centre. We have tried within this analysis to improve the transferability of the results to the wider NHS and other care providers by estimating costs using nationally reported unit costs and accounting for potential variation in costs to different providers. However, it was necessary to use some estimates of costs taken from the single delivery site. Local variation in practice, for example referral rates to secondary care or procedures for avoiding inpatient admissions, can greatly influence the cost of providing an intervention. Analysis of data from a larger multi-centre trial would help address these limitations.

### Benefits over time

Often the pragmatic design of clinical trials limits the follow-up period for data collection. The most-appropriate duration of follow-up for clinical outcomes may differ from those outcomes needed to estimate the long-term cost-effectiveness of an intervention, even if the observed cost-effectiveness ratio is trending towards that of the long-term ICER [34]. It would be ideal, though usually impractical, to measure any benefits accrued over the life-time of the participant. In reality, assumptions about longer term outcomes must usually be made.

In this trial, the three month follow-up period and the wait list design were enforced for ethical reasons and data collected are unlikely to reflect the overall true outcomes for participants with respect to health related quality of life. It is also unlikely that those who experienced benefit at the end of the study period will lose the entirety of that benefit the moment treatment stops. What is not known is for how long benefit is maintained and at what rate it is lost (if at all) over time. In those with advanced cancer, these factors are likely to be influenced by familiarity with the hospice out-patient service, confidence in the probable responsiveness of the clinical team should the patient re-present to the service and deterioration in clinical condition. We have attempted to address this through a series of scenario analyses that extend treatment benefit to a maximum of one year. Though this approach has obvious limitations based on the non-availability of data, it provides decision makers with information derived from modelling scenarios likely to occur beyond the three month trial analysis period and may aid them in considering whether or not to implement the intervention.

### Alternative treatment options

An additional limitation of the trial from the perspective of the economic analyst is that it compares only two alternative treatment modalities. In practice, the particular form of the intervention as practised in the trial is not the only possible way to organise and deliver rehabilitation services for patients living with advanced cancer. In determining the effectiveness from a clinical standpoint, this is appropriate. However it reduces the information available to decision makers who must determine not just whether to offer rehabilitation services but how. Ideally, any future economic evaluation would examine not just the results of this trial but also include evidence from any other trials or sources of robust evidence of day therapy rehabilitation services. However, no other such services have been tested in an RCT, nor do any services have robust evidence of effectiveness and so at present, this evaluation represents the best possible evidence available to decision makers. In Table 4 we provide some evidence of the expected mean cost for each category of resource used in the analysis. Service planners may be able to use such evidence to develop local day therapy rehabilitation services.

### Research considerations for economic evaluations

Caution is required when comparing individual resource use as in Table 4, as the study population was not sufficiently large to make such comparisons, nor was resource use a designated outcome of the trial. Any larger multi-centre study of this intervention should examine such potential resource use related outcomes for participants. It may be useful to explore which component of the intervention provides the most benefits for its cost. In practice, for such complex interventions the individual effect of each component may not be evaluable separately from the overall effect. In a larger study it would be possible to explore whether there are participant sub-groups which might be more likely to benefit from the intervention. An exploratory analysis of our results suggests that women and those with lymphoma reported more benefits than other participants, although these findings are limited by a small sample size.

Another potential concern in economic evaluations is the risk of bias arising from the use of patient recall methods for collecting data on service use. Patient recall methods are commonly used to collect data on patient service use as part of clinical trials and other prospective studies. Concern exists that patients may not be able to recall with sufficient accuracy their contacts with health services over time [35]. Although the evidence base remains limited [35], some research has shown that recall periods of up to six months accurately capture patient resource use [27] The primary alternative to recall methods is the use a patient diary in which study participants record health service use on an on-going basis during the trial period. However, this method is not typically recommended, as respondent burden is considered high and the rates of completion are often low [36].

### Challenges for health care providers

Both the range of ICERs and the CEAC for the base-case analysis indicate uncertainty for decision makers who must choose whether or not the intervention is a cost-effective use of scarce NHS resources. The within-trial analysis, based on costs and outcomes measured at three months, shows that the intervention sits on the margins of cost-effectiveness. This leaves decision makers with a good deal of uncertainty – it is as likely as not that the intervention is cost-effective if there is a willingness to pay of £20,000 per QALY. When the length of time that recipients might expect to maintain benefit from the intervention is extended the ICER decreases, though the probability that the intervention is cost-effective increase only slightly. This reflects the considerable degree of uncertainty in the trial data resulting from the small sample size.

When there is such uncertainty over the likely cost-effectiveness of an intervention decision makers are faced with the question of whether or not to implement the intervention [37]. A number of factors beyond the ICER and CEAC results can influence this decision. These might include the ways in which a technology or intervention is delivered, what recipient groups are likely to be affected by adoption and questions of equity – that is, whether any recipient groups might be unfairly disadvantaged by the decision to adopt an intervention [38,39]. In the case of the intervention studied here, decision makers may be helped by further data on the value of treatment to recipient sub-groups and the ways in which the intervention can be delivered in different settings. In addition decision makers must consider the irreversibility of their decision and the consequences of a wrong decision [40]. In the case of a day therapy rehabilitation service, the establishment of such a service is likely to have few irreversible consequences in circumstances where capital investment requirements are likely to be low and staff training requirements minimal as specialist multi-disciplinary practitioners are usually available within hospice settings. Services could be reduced easily if further research shows that treatment is not cost-effective and resources redirected with relative ease.

## Conclusion

Our analyses reveal uncertainty about whether the rehabilitation intervention represents a cost-effective use of health care resources when compared with usual care. Although commissioners may wish to consider introducing such day therapy services in view of the clinical benefits to patients, they should be mindful of the need for further evaluation of cost-effectiveness. This should be weighed against a) the potential benefits to recipients and b) the relative ease with which a decision could be reversed if the model used follows closely that used in the RCT on which this evaluation is based [30]. Where a day therapy service is introduced formal evaluation should be considered. Value of information analysis would help determine the benefits and inform the design of such an evaluation.
